# Gender-Specific Association of* ATP2B1* Variants with Susceptibility to Essential Hypertension in the Han Chinese Population

**DOI:** 10.1155/2016/1910565

**Published:** 2016-01-11

**Authors:** Jin Xu, Hai-xia Qian, Su-pei Hu, Li-ya Liu, Mi Zhou, Mei Feng, Jia Su, Lin-dan Ji

**Affiliations:** ^1^Department of Preventive Medicine, School of Medicine, Ningbo University, Ningbo 315211, China; ^2^Department of Research and Teaching, Ningbo No. 2 Hospital, Ningbo 315010, China; ^3^Department of Gerontology, Ningbo No. 1 Hospital, Ningbo 315010, China; ^4^Department of Biochemistry, School of Medicine, Ningbo University, Ningbo 315211, China

## Abstract

Previous genome-wide association studies (GWASs) found that several* ATP2B1* variants are associated with essential hypertension (EHT). But the “genome-wide significant”* ATP2B1 *SNPs (rs2681472, rs2681492, rs17249754, and rs1105378) are in strong linkage disequilibrium (LD) and are located in the same LD block in Chinese populations. We asked whether there are other SNPs within the* ATP2B1* gene associated with susceptibility to EHT in the Han Chinese population. Therefore, we performed a case-control study to investigate the association of seven tagSNPs within the* ATP2B1* gene and EHT in the Han Chinese population, and we then analyzed the interaction among different SNPs and nongenetic risk factors for EHT. A total of 902 essential hypertensive cases and 902 normotensive controls were involved in the study. All 7 tagSNPs within the* ATP2B1* gene were retrieved from HapMap, and genotyping was performed using the Tm-shift genotyping method. Chi-squared test, logistic regression, and propensity score analysis showed that rs17249754 was associated with EHT, particularly in females. The MDR analysis demonstrated that the interaction of rs2070759, rs17249754, TC, TG, and BMI increased the susceptibility to hypertension. Crossover analysis and stratified analysis indicated that BMI has a major effect on the development of hypertension, while* ATP2B1* variants have a minor effect.

## 1. Introduction

Because of its high prevalence and substantial impact on several cardiovascular diseases, hypertension is considered a major contributor to the global health burden [1]. Approximately 95% of hypertensive patients are diagnosed with essential hypertension (EHT), which is defined as high blood pressure (BP) with no identifiable cause [2]. EHT is one of the most common complex genetic disorders, with heritability ranging from 31% to 68% [3]. However, attempts to identify the genetic basis of EHT have been frequently unsuccessful and of relatively low yield [4]. The inability to identify the genetic basis of EHT may be due to the cumulative impact of multiple genes interacting with a variety of environmental factors in the pathogenesis of hypertension [5, 6].

In 2009, based on a genome-wide association study (GWAS) conducted by the Cohorts for Heart and Aging Research in Genome Epidemiology (CHARGE) Consortium, genetic polymorphisms of* ATP2B1* were found to be significantly related to systolic blood pressure (SBP), diastolic blood pressure (DBP), and hypertension [7]. These SNPs were also replicated in the European populations by the Global Blood Pressure Genetics (Global BPgen) Consortium [8]. Moreover, combined analysis of these two datasets further confirmed that only* ATP2B1* variants reached genome-wide significance threshold (*P* < 5 × 10^−8^) with SBP (rs2681492), DBP (rs2681472), and hypertension (rs2681472) [9]. Similarly, in a study of the Korean Association Resource (KARE), rs17249754, which is located near the* ATP2B1* gene, was found to be strongly associated with SBP [10]. Moreover, in a study by the Japanese Millennium Genome Project, another* ATP2B1* variant, rs11105378, was found to have the most significant association with hypertension (*P* = 4.1 × 10^−11^), and the association was cross-validated by replication analysis with the Global BPgen dataset (*P* = 5.9 × 10^−4^) [1]. Meta-analysis of GWASs in East Asians indicated that rs17249754 was associated with SBP (*P* = 7.7 × 10^−20^) and DBP (*P* = 1.9 × 10^−13^) [11].

**Figure 1:**
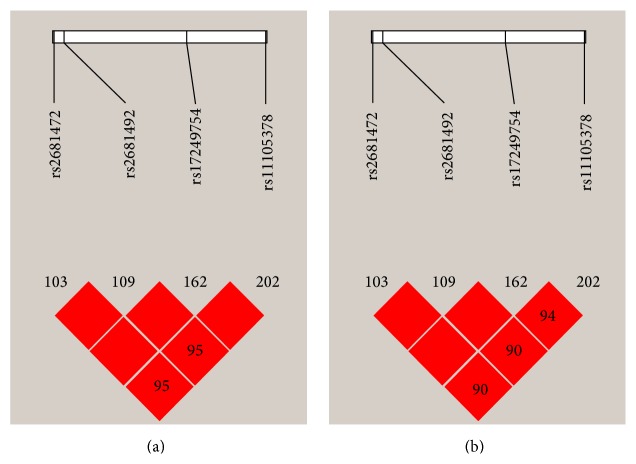
The patterns of linkage disequilibrium for 4 SNPs with *D*′ (a) and *r* ^2^ (b).

Although* ATP2B1* was confirmed to be associated with blood pressure or hypertension in various populations, the “significant” SNPs (rs2681472, rs2681492, rs17249754, and rs1105378) found in the GWASs are in strong linkage disequilibrium (LD) and are located in the same LD block (HapMap CHB *D*′ > 0.95, *r* ^2^ > 0.9) in the Chinese population (Figure 1). We wondered whether there are other SNPs within the* ATP2B1* gene associated with the susceptibility to EHT in the Han Chinese population. In the current study, we conducted a replication analysis to test the association of seven tagSNPs within the* ATP2B1* gene and EHT in the Han Chinese population. Subsequently, we analyzed the interaction among different SNPs and nongenetic risk factors for EHT, which provided additional information on the role of* ATP2B1* variants.

## 2. Materials and Methods

### 2.1. Ethics Statement

The protocol of this study was approved by the medical ethics committee of Ningbo University. The health records and blood samples of the participants were collected with informed written consent.

### 2.2. Study Participants

The details of the study participants have been described previously [12]. Briefly, we collected more than 10,000 health records from our established database of Ningbo Chronic Diseases Cohort. The participants in this database are 30 to 75 years old, Han Chinese, living in Ningbo City (East coast of China) for at least three generations without migration history. Patients with secondary hypertension, severe cardiovascular diseases, diabetes, kidney diseases, or other major chronic illnesses according to their health records were excluded before case-control paring. Hypertension in this study was defined as sitting systolic blood pressure (SBP) ≥140 mmHg and/or diastolic blood pressure (DBP) ≥90 mmHg or self-reported use of antihypertensive medication. Participants with SBP ≤120 mmHg and DBP ≤80 mmHg were recruited as controls. Subsequently, 902 hypertensive cases and 902 normotensive controls, matched for age and sex, were selected with informed consent.

### 2.3. Measurement of Clinical Parameters

With informed written consent, two milliliters of venous blood was collected with ethylene diamine tetraacetic acid as an anticoagulant. Subsequently, the serum levels of total cholesterol (TC), high-density lipoprotein (HDL), and triglyceride (TG) were measured enzymatically using a Hitachi automatic biochemistry analyzer 7100. Clinical information, including body mass index (BMI), and weekly alcohol and cigarette consumption were also obtained. In this study, people who consumed ≥70 g of alcohol per week for more than 1 year were defined as individuals with alcohol abuse. Moreover, people who smoked ≥70 cigarettes per week for more than 1 year were defined as individuals with a smoking habit.

### 2.4. SNP Genotyping

All 7 tagSNPs were retrieved from HapMap using the tagger pairwise method in CHB as follows: *R* ^2^ cutoff = 0.8 and minor allele frequency (MAF) cutoff = 0.1. Genomic DNA was extracted from whole blood through the standard phenol-chloroform method. Genotyping was performed using the Tm-shift genotyping method [13]. To confirm the genotyping results, 100 samples were randomly selected and sequenced with bidirectional coverage by BGI Tech Solutions Company.

### 2.5. Statistical Analysis

Continuous variables are presented as the mean ± SD and analyzed by *t*-test between two groups. Statistical analyses of the allele frequencies between the hypertensive and normotensive subjects and between males and females were performed using the chi-squared test. Logistic regression was used to control the confounding variables. *P* values, odds ratios (ORs), and 95% confidence intervals (CIs) were calculated using SPSS 18.0 (SPSS Inc., Chicago, IL, USA). The propensity score analysis was performed using STATA 13.0 according to the method described by Rosenbaum and Rubin [14]. The Hardy-Weinberg equilibrium (HWE) test for genotype distribution was performed for the controls using PEDSTATS [15]. Multifactor dimensionality reduction (MDR), stratified analysis, and crossover analysis were used to identify and characterize interactions among SNPs and nongenetic factors [16]. *P* values were adjusted for the total number of tested SNPs using the Bonferroni correction method (*α* = 0.05/7 ≈ 0.0071).

## 3. Results

Table 1 shows the baseline characteristics of the participants. Each group consists of 390 males and 512 females, and the mean ages of the hypertensive participants and controls were similar, demonstrating that the case and control groups were well matched. Serum levels of TC and TG and BMI were significantly higher in the hypertensive groups than those in the control group (*P* < 0.01). However, the serum level of HDL and the percentage of participants with a smoking habit or alcohol abuse were not different between two groups.

Table 2 shows the genotypes of each SNP. The success rate of genotyping was 99%, and all SNPs did not deviate from HWE (*P* > 0.05). Based on the prevalence, OR, and MAF in this study, the genetic power calculator indicated that the sample size is large enough to perform a case-control analysis with 80% power [17]. According to the *P* values and ORs, only G allele of rs17249754 is associated with EHT (*P* = 0.005, OR (95% CI) = 1.21 (1.06–1.39)) after correction for multiple testing. However, rs2070759, rs3741895, rs2854371, rs11105357, rs957525, and rs11105358 were not associated with EHT. Inputting all covariates including age, gender, HDL, TC, TG, BMI, smoking habit, and alcohol abuse, the propensity score analysis indicated that still only G allele of rs17249754 is associated with EHT (*P* = 0.007, OR = 1.21). After control of confounding variables including TC, TG, and BMI, logistic regression also confirmed rs17249754 is associated with EHT (*P* = 0.007, OR = 1.21).

Considering gender difference in EHT [18], the genotyping results were further stratified by gender. Interestingly, both the A allele of rs2070759 and the G allele of rs17249754 were significantly associated with EHT only in women (for rs2070759, *P* = 0.008, OR (95% CI) = 1.27 (1.06–1.51); for rs17249754, *P* = 0.017, OR (95% CI) = 1.25 (1.04–1.49)).

MDR was used to analyze the interaction among SNPs and nongenetic risk factors for EHT, and the software output the best model for “BMI” and “rs2070759, rs17249754, TG, TC, and BMI” with 10/10 cross-validation consistency (Table 3). To determine the manner in which BMI and* ATP2B1* variants interact to cause hypertension, we performed a stratified analysis. The result showed that when BMI ≥25, neither SNP is associated with hypertension (*P* > 0.05). However, when BMI <25, the A allele of rs2070759 or the G allele of rs17249754 showed a significant association with hypertension (Table 4), indicating that BMI has a major effect and that the* ATP2B1* variants have minor effects. Additional crossover analysis also confirmed that BMI had the primary effect (Table 5).

## 4. Discussion

Although dozens of GWASs have been conducted to identify genetic markers for BP traits or hypertension over the past two decades,* ATP2B1* may be the first gene that has been cross-validated in different GWASs. The present study confirmed* ATP2B1* variant rs17249754 as strong susceptibility for EHT in the Han Chinese population. The SNP rs17249754 is associated with BP variation and EHT based on several GWASs in different ethnic populations [1, 10, 11, 19], which is also in strong linkage disequilibrium with other genome-wide significant SNPs, such as rs2681472, rs2681492, and rs1105378, within the* ATP2B1* gene. Similar findings in different ethnic groups further strengthen the hypothesis that the* ATP2B1 *gene is a susceptibility locus of likely global significance for BP variation and the development of hypertension.

The* ATP2B1* gene encodes the plasma membrane calcium ATPase isoform 1, which plays a critical role in intracellular calcium homeostasis due to its capacity for removing bivalent calcium ions from eukaryotic cells against very large concentration gradients [20]. Although the pathophysiological implications of* ATP2B1* gene on the development of hypertension are still unclear, results from* ATP2B1* knockout mouse studies suggested that ATP2B1 may play an important role in the regulation of BP through alterations of calcium handling and vasoconstriction in vascular smooth muscle cells [21].* ATP2B1* mRNA expression levels in umbilical artery smooth muscle cells were found to be significantly different among rs11105378 genotypes, which may be a potential mechanism by which changes in the* ATP2B1* gene product levels are involved in BP regulation [1]. According to HapMap CHB, rs17249754 and rs1105378 are in strong linkage disequilibrium (*D*′ = 1, *r* ^2^ = 0.95) in Chinese populations; therefore, rs17249754 was genotyped instead of rs1105378 in the present study. In our replication study, we also found that rs1105378 is significantly associated with hypertension (*P* < 0.01). Therefore, the SNPs rs2681472, rs2681492, and rs17249754 are in strong linkage disequilibrium with rs1105378 and may be a genetic marker for the development of hypertension, whereas rs1105378 may have a biological function.

Another finding of the present study is that* ATP2B1* variants are associated with EHT only in women. According to the World Health Organization's (WHO) “Global Status Report on Noncommunicable Diseases 2014” (http://www.who.int/nmh/publications/ncd-status-report-2014/en/), hypertension occurs at a lower rate and at a later age in females than males in all WHO regions. The impact of gender on the prevalence, presentation, and long-term outcome of hypertension has long been a topic of active research. Recent data from several large epidemiological studies showed that awareness, treatment, and control rates of hypertension are higher among women than men, which may cause the gender difference in hypertension [22, 23]. The pathophysiological mechanisms underlying the disparity in blood pressure levels between the two genders are poorly defined, although many hypotheses have been proposed, with hormonal hypotheses prevailing [24]. Similar to our study, several previous studies also found a gender-specific association between gene polymorphisms and EHT [25–27]. Therefore, further basic research is of paramount importance to uncover the genetic and biological mechanisms mediating potential gender differences in hypertension.

EHT is a typical complex disease [28], with dozens of risk factors, such as obesity, physical inactivity, high-fat diet, cigarette smoking, alcohol abuse, excessive salt intake, and mental stress [29–31]. Growing evidence indicates that interactions among multiple genes and environmental factors may increase the susceptibility to EHT [32]. Our previous study has shown that interaction analysis may provide somewhat more information than a single genetic association study [12, 33]. In the present study, MDR analysis demonstrated that BMI itself and the interaction between* ATP2B1* variants and BMI increase the susceptibility to hypertension. Because BMI represents the internal metabolic status and physiological environment [34], it is not surprising that BMI has a major effect in the development of hypertension, while the* ATP2B1* variants have a minor effect. With the development of statistical methods for the evaluation of gene-gene and gene-environment interactions, more missing inheritability will be identified and more specific mechanisms will be discovered [35, 36].

In conclusion, we confirmed the association of* ATP2B1* variants with the susceptibility to EHT in the Han Chinese population, especially in the females. Moreover, the interaction of BMI and* ATP2B1* variants increased the susceptibility to hypertension, with BMI having a major effect and* ATP2B1* variants having a minor effect.

## Figures and Tables

**Table 1:** Baseline characteristics of the investigated participants.

Variables	Case	Control	*P* value
Number	902	902	N/A
Male/female	390/512	390/512	N/A
Age (y)	56.92 ± 7.36	56.59 ± 7.43	*P* = 0.45
TG (mM)	2.02 ± 1.68	1.64 ± 1.12	*P* < 0.01
HDL (mM)	1.41 ± 0.35	1.41 ± 0.33	*P* = 0.71
TC (mM)	5.33 ± 1.01	5.18 ± 0.93	*P* < 0.01
BMI (Kg/m^2^)	24.65 ± 3.25	23.22 ± 2.88	*P* < 0.01
Smoking habit	171	147	*P* = 0.14
Alcohol abuse	152	148	*P* = 0.80

TG: triglyceride; HDL: high-density lipoprotein; TC: total cholesterol; BMI: body mass index.

**Table 2:** Association statistics for the *ATP2B1* variants and hypertension.

SNP	Genotype	Group	Genotype			MAF	*P* value	OR	95% CI
rs3741895	AA/AG/GG	Case	778	122	0	0.07	0.954	0.99	0.77–1.29
		Control	778	121	0	0.07			
		Male case	331	58	0	0.07	0.433	0.86	0.58–1.27
		Male control	338	50	0	0.06			
		Female case	447	64	0	0.06	0.533	1.12	0.79–1.59
		Female control	440	71	0	0.07			

rs2854371	CC/CT/TT	Case	519	339	44	0.24	0.879	1.01	0.87–1.18
		Control	510	347	41	0.24			
		Male case	226	143	21	0.24	0.648	0.95	0.75–1.20
		Male control	226	146	15	0.23			
		Female case	293	196	23	0.24	0.553	1.06	0.87–1.30
		Female control	284	201	26	0.25			

rs2070759	AA/AC/CC	Case	266	453	183	0.45	0.036^*∗*^	1.15	1.01–1.31
		Control	223	476	203	0.49			
		Male case	107	201	82	0.47	0.879	1.02	0.83–1.24
		Male control	109	194	87	0.47			
		Female case	159	252	101	0.44	0.008^*∗*^	1.27	1.06–1.51
		Female control	114	282	116	0.50			

rs11105357	CC/CT/TT	Case	722	172	8	0.10	0.472	0.92	0.74–1.15
		Control	733	163	6	0.10			
		Male case	320	64	6	0.10	0.407	1.15	0.83–1.59
		Male control	309	76	5	0.11			
		Female case	402	108	2	0.11	0.088	0.78	0.58–1.04
		Female control	424	87	1	0.09			

rs957525	AA/AG/GG	Case	546	314	40	0.22	0.705	1.03	0.88–1.21
		Control	541	313	45	0.22			
		Male case	245	132	12	0.20	0.239	1.16	0.91–1.48
		Male control	231	141	17	0.22			
		Female case	301	182	28	0.23	0.615	0.95	0.77–1.17
		Female control	310	172	28	0.22			

rs11105358	CC/CG/GG	Case	43	311	547	0.22	0.160	1.12	0.96–1.32
		Control	35	293	574	0.20			
		Male case	24	124	242	0.22	0.903	1.02	0.80–1.29
		Male control	18	134	238	0.22			
		Female case	19	187	305	0.22	0.076	1.22	0.98–1.51
		Female control	17	159	336	0.19			

rs17249754	AA/AG/GG	Case	102	417	383	0.34	0.005^*∗*^	0.82	0.72–0.94
		Control	143	416	343	0.39			
		Male case	46	182	162	0.35	0.128	0.85	0.69–1.05
		Male control	59	185	146	0.39			
		Female case	56	235	221	0.34	0.017^*∗*^	0.80	0.67–0.96
		Female control	84	231	197	0.39			

*P* values were obtained from the comparison of two allele frequencies. OR: odds ratio; CI: confidence interval. ^*∗*^ *P* value was less than 0.05.

**Table 3:** MDR analysis of gene-environment interaction.

Best model	Testing odds ratio	Testing *X* ^2^	Cross-validation consistency
BMI	2.25 (95% CI: 1.19–4.24)	6.36 (*P* = 0.012)	10/10
BMI, TG	2.00 (95% CI: 1.10–3.61)	5.27 (*P* = 0.021)	9/10
rs2070759, rs17249754, TG, TC, and BMI	1.83 (95% CI: 1.01–3.30)	4.05 (*P* = 0.044)	10/10

**Table 4:** Stratified analysis of interaction between BMI and *ATP2B1* variants.

SNP	Genotype	BMI	Group	Number			*P* value	OR	95% CI
rs2070759	AA/AC/CC	<25	Case	147	268	104	0.022^*∗*^	1.21	1.03–1.42
			Control	156	359	164			
		≥25	Case	115	185	83	0.349	0.89	0.71–1.13
			Control	70	114	39			

rs17249754	AA/AG/GG	<25	Case	59	241	219	0.011^*∗*^	0.80	0.68–0.95
			Control	104	331	244			
		≥25	Case	43	176	164	0.61	0.94	0.74–1.20
			Control	37	85	101			

*P* values were obtained from the comparison of two allele frequencies. OR: odds ratio; CI: confidence interval.

^*∗*^ *P* value was less than 0.05.

**Table 5:** Crossover analysis of interaction between BMI and *ATP2B1* variants.

SNP	BMI	Allele	Case	Control	*P* value	OR	95% CI
rs2070759	<25	C	476	687	1	1	NA
	<25	A	562	671	0.022^*∗*^	1.21	1.03–1.42
	≥25	C	351	192	*P* < 0.001^*∗*^	0.38	0.31–0.47
	≥25	A	415	254	*P* < 0.001^*∗*^	0.42	0.35–0.52

rs17249754	<25	A	359	539	1	1	NA
	<25	G	679	819	0.011^*∗*^	0.80	0.68–0.95
	≥25	A	262	159	*P* < 0.001^*∗*^	0.40	0.32–0.51
	≥25	G	504	287	*P* < 0.001^*∗*^	0.38	0.31–0.46

*P* values were obtained from the comparison of two allele frequencies. OR: odds ratio; CI: confidence interval.

^*∗*^ *P* value was less than 0.05.
